# Variability of nursing care by APR-DRG and by severity of illness in a sample of nine Belgian hospitals

**DOI:** 10.1186/1472-6955-12-26

**Published:** 2013-10-10

**Authors:** Magali Pirson, Caroline Delo, Lionel Di Pierdomenico, Nancy Laport, Veronique Biloque, Pol Leclercq

**Affiliations:** 1 Health Economics, Hospital’s Management and Nursing research Department, CP592- School of Public Health, Université Libre de Bruxelles, 808, Route de Lennik, B- 1070, Bruxelles, Belgium

**Keywords:** Nursing cost, DRG, Financing systems, Outliers

## Abstract

**Background:**

As soon as Diagnosis related Groups (DRG) were introduced in many hospital financing systems, most nursing research revealed that DRG were not very homogeneous with regard to nursing care. However, few studies are based on All Patient refined Diagnosis related Groups (APR-DRGs) and few of them use recent data. Objectives of this study are: (1) to evaluate if nursing activity is homogeneous by APR-DRG and by severity of illness (SOI) (2) to evaluate the outlier’s rate associated with the nursing activity and (3) to compare nursing cost homogeneity per DRG and SOI.

**Methods:**

Study done in 9 Belgian hospitals on a selection of APR-DRG with more than 30 patients (7 638 inpatient stays). The evaluation of the homogeneity is based on coefficients of variation (CV). The 75th percentile + 1.5 × inter-quartile range was used to select high outliers. 25th percentile −1.5 × inter-quartile range was used to select low outliers. Nursing costs per ward were distributed on inpatient stays of each ward following two techniques (the LOS vs. the number of nursing care minutes per stay).

**Results:**

The homogeneity of LOS by DRG and by SOI is relatively good (CV: 0.56). The homogeneity of the nursing activity by DRG is less good (CVs between 0.36 and 1.54) and is influenced by nursing activity outliers (high outliers’ rate: 5.19%, low outliers’ rate: 0.14%). The outlier’s rate varies according to the studied variable. The high outliers’ rate is higher for nursing activity than for LOS. The homogeneity of nursing costs is higher when costs are based on the LOS of patients than when based on minutes of nursing care (CVs between 0.26 and 1.46 for nursing costs based on LOS and between 0.49 and 2.04 for nursing costs based on minutes of nursing care).

**Conclusions:**

It is essential that the calculation of nursing cost by stay and by DRG for hospital financing purposes was based on nursing activity data, that more reflect resources used in wards, and not on LOS data. The only way to obtain this information is the generalization of computerized nursing files.

## Background

### The Belgian context

The Belgian hospital funding system for inpatients combines different elements: a global budget system (“Budget of Financial Means”) (BMF) for administrative costs, hotel costs, investments, nursing care, etc. (42-45%); a combination of a fee for service system and lump sums for medical procedures (42-45%); the reimbursement of drugs (partially based on lump sums), prosthesis, blood (12-15%) [1].

Nursing care is mainly financed by the BMF. The biggest part of the BMF is calculated on the basis of “All Patient refined diagnosis related groups” (APR-DRGs) through “justified” days, that means national length of stay (LOS) by disease. About 6.5% of the national budget is reserved for supplementing the BMF of each hospital with an additional budget allocation, partly based on nursing interventions data [2].

For this supplement, hospitals record nursing activity four times a year (4*15 days). The Belgian Nursing Minimum Data Set (NMDS) was composed of 23 items until 2006 and 78 items from 2008, selected from the Nursing Interventions Classification (NIC). The NMDS is used to classify inpatient days into 28 zones, with a weight based on actual nurse staffing level (number and qualification level). The additional budget allocation is based on the number of inpatient days per zone and their weight [3].

### The international context and objectives of the study

It is common in Europe, as well as in United States (US) for hospitals to be funded through the implementation of prospective payment systems using diagnosis related groups (DRGs) [4]. DRGs characterize groups of patients based on economic and clinical homogeneity. Clinical homogeneity is achieved on the basis of medical diagnosis, co-morbidities, medical procedures, complications, etc. Economic homogeneity is achieved by using first of all the LOS or cost (or charges) of hospitalization as classification criterion. In this kind of financing system, a tariff is fixed in advance for each DRG [5].

As soon as DRGs were introduced, most nursing research revealed that DRG were not very homogeneous with regard to nursing care [6-10].

Some researchers argued that the shortened length of stay in hospitals has created greater variability in the nursing care needs by inpatients [10].

Some countries have introduced nursing intensity adjustment to correct DRG payment and reflect nursing care differences. For example, in New York, nursing costs are allocated to each DRG in payment rate formulation by means of nursing intensity weights (NIWs) relative values reflecting the quantity and types of nursing services provided to patients in each DRG [5]. The methods either use standardized set of nursing intensity weights for each DRG or measure levels of nursing care using nursing classification data which can be used to adjust billing [11]. Research also shows that DRG only explain 20% to 40% in the variability of nursing care. Coefficients of variation for nursing care per DRG are reported varying from 0.22 to 2.56 [12-16].

In Belgium, some researchers try also to refine DRGs classification into classes of nursing cost per DRG [3].

There are a lot of publications regarding the heterogeneity of nursing care inside DRG [16] but few of them are based on APR-DRG and few of them study recent data.

The evaluation of the homogeneity of nursing care by DRG can be done on nursing activity and on nursing cost. Studies showed that various results on nursing costs were due to different methods and study samples employed. Most studies are based on one single hospital, some are done across hospitals. Some of studies calculate nursing as a percentage of hospital charges, while others express nursing as a percentage of hospital expenditures. Methodologies can also be different to evaluate costs per DRG (ratio of cost-to charges, relative value units, nursing intensity weight, etc [17]. This last method consists to estimate the level of intensity of nursing workload for each DRG on a per diem basis through interactive process utilizing an expanded approach to Delphi iteration and feedback from a nursing panel.

Others methodologies can be used: the calculation of an average nursing cost per patient stay in which the cost of nursing care is directly related to the number of inpatient days or a method based on the calculation of a nursing cost directly related to nursing workload. This last method is much more precise.

Objectives of this study are: (1) to evaluate if nursing activity is homogeneous by APR-DRG and by severity of illness (SOI) in a sample of nine Belgian hospitals (2) to evaluate the outlier’s rate associated with the nursing activity and (3) to compare two methodologies of allocation of nursing cost by stay and by DRG.

## Methods

### Description of the sample

9 Belgian hospitals (8 general hospitals and one teaching hospital).

### Representativeness of hospitals

In 2008, the total of inpatient stays from the sample is 107 920. It represents 6,60% of all Belgian inpatient stays (Hospital H1: 6 449 inpatient stays, Hospital H10_F1: 12 700 inpatient stays, Hospital H10_F3: 11 266 inpatient stays, Hospital H2: 12 595 inpatient stays, Hospital H3: 7 212 inpatient stays, Hospital H4: 14 146 inpatient stays, Hospital H5: 8 929 inpatient stays, Hospital H6: 21 821 inpatient stays, Hospital H7: 12 802 inpatient stays).

### Selection of inpatient stays

DRGs and SOI with more than 30 patients were selected. Among these DRGs and SOI, we selected inpatients having a NMDS in 2008 and representing a complete stay (during the federal recording period: 4* 15 days a year). 7 638 inpatient stays were selected according to this criterion.

### Variables: LOS and nursing activity

For the evaluation of nursing activity by stay and disease, NMDS were converted into minutes of nursing care through minutes by item published in two federal reports [18,19]. Inpatients stays were aggregated into DRGs and SOI. LOS has been given per inpatient stay and per medical ward, by hospitals.

### Evaluation of the homogeneity of LOS and nursing activity

The evaluation is based on the coefficient of variation (standard deviation/mean). Coefficients of variation were calculated for each variable, firstly on all patients and secondly on inliers only (because outliers can induce heterogeneity). Coefficients of variation are calculated by DRG and by SOI.

### Selection of outliers (patient level)

The 75th percentile + 1.5 × inter-quartile range was used to select high nursing activity outliers. 25th percentile −1.5 × inter-quartile range was used to select low nursing activity outliers. The same rules were used to select LOS outliers. This selection was done by DRG and by SOI.

### Calculation of a nursing cost by inpatient stay and by DRG and SOI

This calculation was only done in one hospital of the sample, having the NMDS available on a daily basis. Others hospitals only record the NMDS two weeks four times a year. Nursing costs per ward were extracted from the accountancy database, which means that salary costs by ward are taken into account. Nursing costs per ward were distributed on inpatient stays of each ward following two techniques (the LOS vs. the number of nursing care minutes per stay). A suppression of partial stays was carried out after the allocation. Inpatient stays were linked to their respective DRG and SOI. A comparison of costs by inpatient stay according to the methodology used was done. A comparison of CV of nursing costs by pathology, based on the two techniques, was carried out.

Precautions have been taken to protect the privacy of research subjects and the confidentiality of their personal information. Data have been completely anonymized by hospitals before sending to the research team. No access to medical records was performed.

Each hospital has given written permission to analyze these data in the context of research.

## Results

### DRGs and SOI

7 638 inpatients are assigned to 110 groups of DRGs and SOI. 27 groups concentrate 50% of patients.

### Homogeneity of the nursing activity by DRG and by SOI

CVs were calculated by DRG and severity of illness. CVs vary between 0.36 and 1.54. The median of CVs by DRG and severity of illness is 0.75. These values are slightly influenced by nursing activity outliers.

### Outliers rates

The outlier’s rate varies according to the studied variable. The high outliers’ rate is higher for nursing activity than for LOS (Table 1). The high outliers’ rate for nursing activity is 5.19%. The low outliers’ rate is 0.14%. Outliers are more important for several DRG and SOI (Table 2), which means that there are large consumers of nursing care, even in a same DRG and a same severity of index. 27.02% of high nursing activity outliers are also high LOS outliers. 71.97% of high nursing activity outliers are LOS inliers and 1.01% of high nursing activity outliers are low LOS outliers. 27.27% of low nursing activity outliers are also low LOS outliers. 72.73% of low nursing activity outliers are LOS inliers.

**Table 1:** Comparison of outliers rates for nursing activity and LOS

**Data**	**High outliers (%)**	**Low outliers (%)**	**Inliers (%)**
Nursing activity (n = 7 638)	5.19%	0.14%	94.67%
LOS (n = 7 638)	4.77%	0.42%	94.81%

LOS: length of stay.

**Table 2:** DRGs and SOI having a high rate (>10%) of high nursing activity outliers

**DRGSEV**	**DRG**	**N**	**N outliers**	**%**
640_1	640 NEONATE BIRTH WEIGHT > 2499 G. NORMAL NEWBORN OR NEONATE W OTHER PROBLEM	51	9	17.65%
861_1	861 SIGNS & SYMPTOMS	40	7	17.50%
144_2	144 RESPIRATORY SYSTEM SIGNS. SYMPTOMS & OTHER DIAGNOSES	89	15	16.85%
283_1	283 DISORDERS OF LIVER EXCEPT MALIG. CIRRHOSIS OR ALCOHOLIC HEPATITIS	30	5	16.67%
058_1	058 OTHER DISORDERS OF NERVOUS SYSTEM	209	33	15.79%
420_2	420 DIABETES	30	4	13.33%
540_1	540 CESAREAN DELIVERY	81	10	12.35%
144_1	144 RESPIRATORY SYSTEM SIGNS. SYMPTOMS & OTHER DIAGNOSES	59	7	11.86%
225_1	225 APPENDECTOMY	58	6	10.34%
540_2	540 CESAREAN DELIVERY	49	5	10.20%
201_1	201 CARDIAC ARRHYTHMIA & CONDUCTION DISORDERS	50	5	10.00%
563_1	563 THREATENED ABORTION	50	5	10.00%
283_2	283 DISORDERS OF LIVER EXCEPT MALIG. CIRRHOSIS OR ALCOHOLIC HEPATITIS	30	3	10.00%
316_1	316 HAND & WRIST PROCEDURES	30	3	10.00%

DRGSEV: DRG_severity of illness.

### Homogeneity of the nursing activity by DRG and by SOI for inliers only

Once having extracted these outliers, the homogeneity by DRG and SOI improves (Table 3) but is nevertheless worse than when based on the LOS. The homogeneity of LOS by DRG and by SOI is relatively good. Average CV (0.56) and median CV (0.56) are lower for LOS than for minutes of care by DRG and SOI. On a sample of 102 DRG and SOI, and after having extracted outliers, 2 DRG and SOI have a CV higher than 1 and 82 DRG and SOI have a CV higher than 0.5. Table 4 shows DRGs having the higher heterogeneity for nursing activity.

**Table 3:** Coefficients of variation for LOS and nursing activity by DRG and by SOI

**Variables**	**Max**	**Min**	**Mean**	**Med**
NURSING ACTIVITY (all patients)	1.54	0.36	0.80	0.75
NURSING ACTIVITY (INLIERS)	1.24	0.18	0.61	0.60
LOS (all patients)	1.19	0.05	0.56	0.56
LOS (INLIERS)	0.75	0.04	0.45	0.46

Max: the higher CV.

Min: the lower CV.

Mean: the average CV.

Med: the median CV.

**Table 4:** DRGs having the higher heterogeneity for nursing activity (inliers only)

**DRG and SOI**	**Name of DRG**	**Mean of nursing minutes per stay**	**SD**	**CV**	**Number of patients**
760_1	OTHER MENTAL DISORDERS	63.48	78.47	1.24	64
144_1	RESPIRATORY SYSTEM SIGNS. SYMPTOMS & OTHER DIAGNOSES	165.20	181.75	1.10	52
058_2	OTHER DISORDERS OF NERVOUS SYSTEM	93.85	92.75	0.99	148
058_1	OTHER DISORDERS OF NERVOUS SYSTEM	39.23	35.90	0.92	176
053_2	SEIZURE	230.66	207.78	0.90	31
566_1	OTHER ANTEPARTUM DIAGNOSES	243.02	201.50	0.83	57
173_2	OTHER VASCULAR PROCEDURES	276.51	228.65	0.83	43
342_1	FRACTURE OR DISLOCATION EXCEPT FEMUR & PELVIS	243.67	201.17	0.83	42
190_2	CIRCULATORY DISORDERS W AMI	451.02	372.33	0.83	33
463_1	KIDNEY & URINARY TRACT INFECTIONS	371.22	294.31	0.79	57

SD: standard deviation.

CV: coefficient of variation.

### Nursing cost by stay and by DRG and SOI

Nursing cost by inpatient stay was calculated in one hospital of the sample. An amount of 6 501 499.24€ of nursing costs has been distributed to 10 472 inpatient stays according to 2 different techniques. The first technique consists of a distribution of nursing costs on the basis of the LOS of patients. The second one distributes nursing costs on the basis of minutes of nursing care per stay. Costs by stay were aggregated by DRG and SOI. The mean cost is 620.85€ for each method used but standard deviations and medians are different: the standard deviation and the median are respectively 918.91€ and 329 by using the minutes of nursing care and 738.45€ and 384.68€ by using the LOS.

The difference by inpatient stay, according to the methodology used, is in average €297.62 by patient (median of €157.58).

The homogeneity of nursing costs by DRG and by SOI improves when costs are based on the LOS of patients. CVs vary between 0.26 and 1.46 for nursing costs based on LOS and between 0.49 and 2.04 for nursing costs based on minutes of nursing care (Table 5).

**Table 5:** Coefficients of nursing cost by DRG and by SOI according to the methodology used (minutes of nursing care vs. LOS)

**CVs of nursing costs**	**Minutes of nursing care**	**LOS**
Max	2.04	1.46
Min	0.49	0.26
Mean	0.92	0.71
Median	0.84	0.65

## Discussion

DRGs are a system for describing the types of patients discharged from acute care hospitals. They were designed to be clinically coherent in the sense that they are expected to evoke a set of clinical responses which result in a similar pattern of resource use for similar groups of patients.

According to Fetter [20], the original development of the DRGs groups had nothing to do with prospective payment. The development of DRGs initially began as an attempt to define operationally the products of a hospital in terms of groups of patients receiving similar sets of outputs or services (such as laboratory tests, x-rays, nursing care). We can thus expect homogeneity of the nursing care by DRG and SOI.

Fetter defines an acceptable level of homogeneity as a ratio of standard deviation to mean of less than one [20]. This statistical threshold is not universally accepted. For example, Fischer [21] defined AP-DRGs with a very high variation of nursing workload when CV > 1 and have a high variation when CV >0.5. According to Atwood et al., the variability is considered high when the coefficient of variation approaches or exceeds the 0.1 [7].

According to the threshold used, conclusions can be very different about the homogeneity of nursing care by DRG and SOI and even about the homogeneity of LOS and cost by DRG and SOI, which is the basis of the DRG system and of all financing systems based on DRGs.

Results of this study show that the homogeneity by DRG and by SOI is higher for LOS than for nursing care.

The homogeneity of nursing cost by DRG and SOI however is not as good as the homogeneity of nursing activity by DRG and by SOI. Nursing costs include resources used in hospitals by ward. Cost by inpatient stay can be very different according to the methodology used. Results should however be confirmed on a broader sample, because the nursing cost study was performed on only one hospital, because other hospitals did not have nursing activity data on a daily basis.

A financing model based on LOS and not on the nursing activity lead to more homogenous groups, because a major part of variation is not measured or taking into account.

Results of this study should be confirmed by realizing another study in all Belgian hospitals and by actualizing data.

## Conclusions

In spite of the weakness of DRGs, a lot of countries use this system to organize their financing system. It is partially the case in Belgium. A recent report [4] explored the feasibility of introducing all-inclusive case-based payments for Belgian hospitals. In a such system, patients are aggregated into APR-DRGs and tariffs have to be set for each APR-DRG. Tariffs, based on weights by DRG can be imported with the grouper or can be developed locally, which is preferable. Tariffs should reflect actual costs, which means that the system should be developed on the basis of detailed cost data. At this moment, no nationwide registration of patient-level costs is available in Belgium. However, ten hospitals (+/− 10% of all hospitals of the country) have this information because they take part in a pilot project in collaboration with the “Université Libre de Bruxelles” (School of Public Health).

However, the usefulness of cost data depends on the methodology used. It is essential that the calculation of nursing cost by inpatient stay and by DRG was based on nursing activity data and not on LOS data which does not reflect the variability of care. The only way to obtain this information is the generalization of computerized nursing files. This recommendation can be done for a lot of countries.

A nationwide cost study should be done in Belgium in a representative sample of hospitals (public, private and teaching hospitals). If results of a such study showed an heterogeneity of nursing cost and total costs by DRG and SOI, it should be necessary to consider a scission or the creation of new groups of DRGs. Another solution is a financing system for nurses, not based on DRG, which is however not the trend in Europe.

## Competing interests

The authors declare that they have no competing interests.

## Authors’ contributions

MP: has made substantial contributions to conception and design, acquisition of data, analysis and interpretation of data. Has have been involved in drafting the manuscript and revising it critically for important intellectual content. Has given final approval of the version to be published. CD: has made substantial contributions to acquisition of data, analysis and interpretation of data. Has been involved in drafting the manuscript and revising it critically for important intellectual content. LDP: has made substantial contributions to acquisition of data, analysis and interpretation of data. Has been involved in revising the manuscript. NL: has made substantial contributions to interpretation of data. Has have been involved in revising the manuscript critically for important intellectual content. VB: has made substantial contributions to acquisition and analysis of data. PL: has made substantial contributions to conception and design, acquisition of data, analysis and interpretation of data. Has have been involved in revising the manuscript critically for important intellectual content. Has given final approval of the version to be published. All authors read and approved the final manuscript.

## Pre-publication history

The pre-publication history for this paper can be accessed here:

http://www.biomedcentral.com/1472-6955/12/26/prepub
